# Supplementary material for: Effectiveness and Safety of Ayurvedic Medicines in Type 2 Diabetes Mellitus Management: A Systematic Review and Meta-Analysis
Source: Front Pharmacol. 2022 Jun 8;13:821810. doi: 10.3389/fphar.2022.821810 (PMC9213670; doi:10.3389/fphar.2022.821810)
Supplement: Supplementary file 1 [file DataSheet1.zip › Supplementary Material/Forest and Funnel Plots/Trigonella foenum-graecum/Subgroup analyses - country/Trigonella foenum-graecum_FBG_country subgroup analysis.pdf]

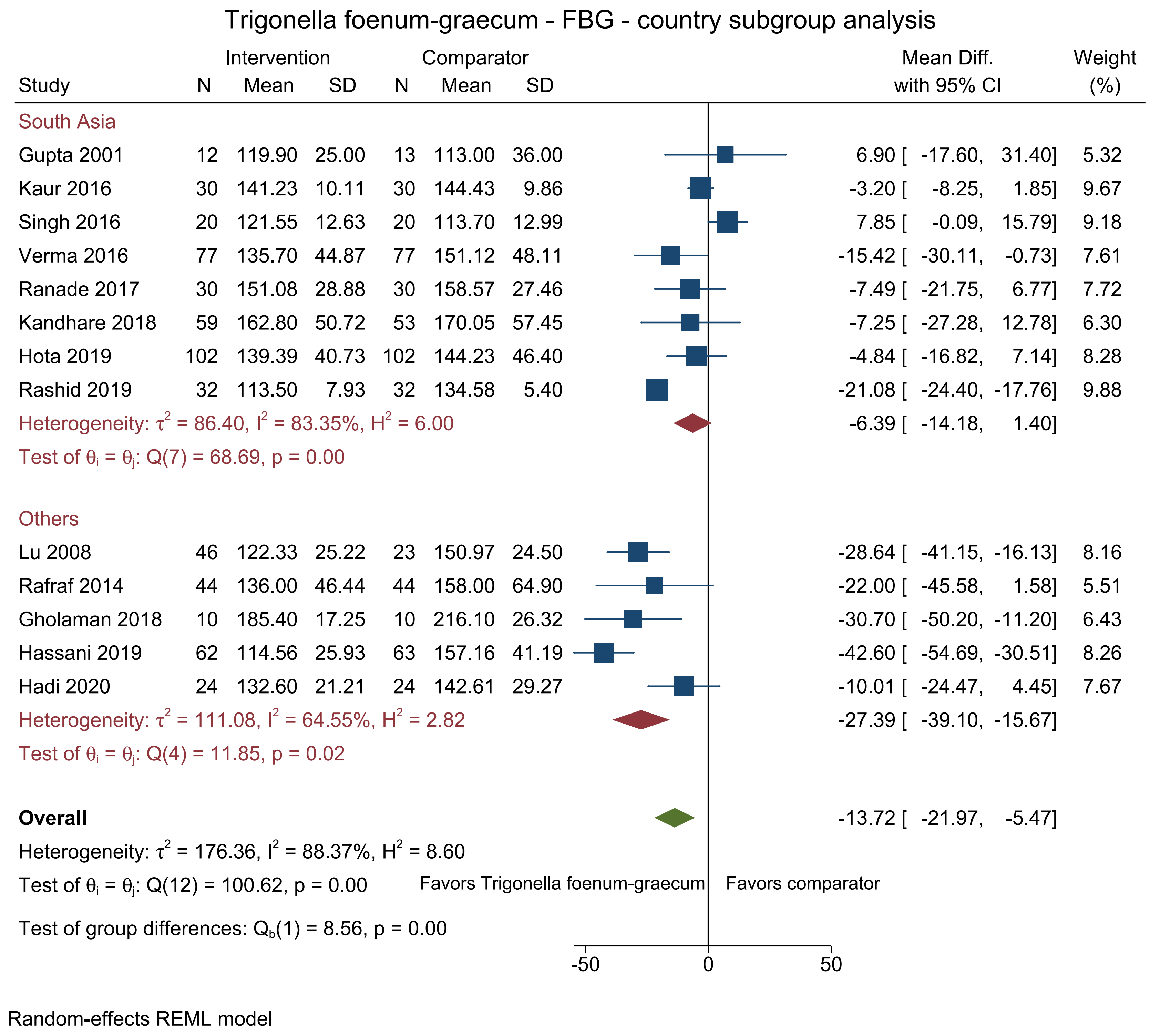

Trigonella foenum-graecum - FBG - country subgroup analysis
Intervention
Comparator
Study
N
Mean
SD
N
Mean
SD
Mean Diff. with 95% CI
Weight (%)
South Asia
Gupta 2001 12 119.90 25.00 13 113.00 36.00 6.90 [-17.60, 31.40] 5.32
Kaur 2016 30 141.23 10.11 30 144.43 9.86 -3.20 [-8.25, 1.85] 9.67
Singh 2016 20 121.55 12.63 20 113.70 12.99 7.85 [-0.09, 15.79] 9.18
Verma 2016 77 135.70 44.87 77 151.12 48.11 -15.42 [-30.11, -0.73] 7.61
Ranade 2017 30 151.08 28.88 30 158.57 27.46 -7.49 [-21.75, 6.77] 7.72
Kandhare 2018 59 162.80 50.72 53 170.05 57.45 -7.25 [-27.28, 12.78] 6.30
Hota 2019 102 139.39 40.73 102 144.23 46.40 -4.84 [-16.82, 7.14] 8.28
Rashid 2019 32 113.50 7.93 32 134.58 5.40 -21.08 [-24.40, -17.76] 9.88
Heterogeneity: τ^2 = 86.40, I^2 = 83.35%, H^2 = 6.00 -6.39 [-14.18, 1.40]
Test of $\theta_i = \theta_j$: Q(7) = 68.69, p = 0.00
Others
Lu 2008 46 122.33 25.22 23 150.97 24.50 -28.64 [-41.15, -16.13] 8.16
Rafraf 2014 44 136.00 46.44 44 158.00 64.90 -22.00 [-45.58, 1.58] 5.51
Gholaman 2018 10 185.40 17.25 10 216.10 26.32 -30.70 [-50.20, -11.20] 6.43
Hassani 2019 62 114.56 25.93 63 157.16 41.19 -42.60 [-54.69, -30.51] 8.26
Hadi 2020 24 132.60 21.21 24 142.61 29.27 -10.01 [-24.47, 4.45] 7.67
Heterogeneity: τ^2 = 111.08, I^2 = 64.55%, H^2 = 2.82 -27.39 [-39.10, -15.67]
Test of $\theta_i = \theta_j$: Q(4) = 11.85, p = 0.02
Overall -13.72 [-21.97, -5.47]
Heterogeneity: τ^2 = 176.36, I^2 = 88.37%, H^2 = 8.60
Test of $\theta_i = \theta_j$: Q(12) = 100.62, p = 0.00
Favors Trigonella foenum-graecum
Favors comparator
Test of group differences: $Q_b(1)$ = 8.56, p = 0.00
-50
0
50
Random-effects REML model
